# FTY720/Fingolimod Reduces Synucleinopathy and Improves Gut Motility in A53T Mice: CONTRIBUTIONS OF PRO-BRAIN-DERIVED NEUROTROPHIC FACTOR (PRO-BDNF) AND MATURE BDNF*

**DOI:** 10.1074/jbc.M116.744029

**Published:** 2016-08-15

**Authors:** Guadalupe Vidal-Martínez, Javier Vargas-Medrano, Carolina Gil-Tommee, David Medina, Nathan T. Garza, Barbara Yang, Ismael Segura-Ulate, Samantha J. Dominguez, Ruth G. Perez

**Affiliations:** From the Center of Emphasis in Neurosciences, Graduate School of Biomedical Sciences, Texas Tech University Health Sciences Center El Paso, Paul L. Foster School of Medicine, El Paso, Texas 79905

**Keywords:** alpha-synuclein (aSyn), neurodegenerative disease, neuroprotection, Parkinson disease, pathology, gut, preclinical assessment, synucleinopathy

## Abstract

Patients with Parkinson's disease (PD) often have aggregated α-synuclein (aSyn) in enteric nervous system (ENS) neurons, which may be associated with the development of constipation. This occurs well before the onset of classic PD motor symptoms. We previously found that aging A53T transgenic (Tg) mice closely model PD-like ENS aSyn pathology, making them appropriate for testing potential PD therapies. Here we show that Tg mice overexpressing mutant human aSyn develop ENS pathology by 4 months. We then evaluated the responses of Tg mice and their WT littermates to the Food and Drug Administration-approved drug FTY720 (fingolimod, Gilenya) or vehicle control solution from 5 months of age. Long term oral FTY720 in Tg mice reduced ENS aSyn aggregation and constipation, enhanced gut motility, and increased levels of brain-derived neurotrophic factor (BDNF) but produced no significant change in WT littermates. A role for BDNF was directly assessed in a cohort of young A53T mice given vehicle, FTY720, the Trk-B receptor inhibitor ANA-12, or FTY720 + ANA-12 from 1 to 4 months of age. ANA-12-treated Tg mice developed more gut aSyn aggregation as well as constipation, whereas FTY720-treated Tg mice had reduced aSyn aggregation and less constipation, occurring in part by increasing both pro-BDNF and mature BDNF levels. The data from young and old Tg mice revealed FTY720-associated neuroprotection and reduced aSyn pathology, suggesting that FTY720 may also benefit PD patients and others with synucleinopathy. Another finding was a loss of tyrosine hydroxylase immunoreactivity in gut neurons with aggregated aSyn, comparable with our prior findings in the CNS.

## Introduction

The chaperone-like protein aSyn^2^ (1, 2) is highly expressed in neurons of the CNS and the peripheral nervous system (PNS) (3, 4). Intraneuronal Lewy bodies, the pathological hallmarks of PD, contain high levels of aggregated aSyn (5). Although rare PD families have aSyn mutations, multiplications, or expansion of the aSyn Rep1 allele (6–14), most PD is sporadic and linked to aging; yet aSyn is abundant in all Lewy bodies, which are present in most cases of PD (15). The typical motor symptoms of PD emerge after an extensive loss of substantia nigra pars compacta dopaminergic neurons (16); however, the so-called pre-motor symptoms arise years earlier (17–19). The discovery of pre-motor symptoms offers hope for early PD diagnosis (20, 21), which could be beneficial as successful neuroprotective therapies emerge.

Constipation is a common symptom that can begin up to 20 years before motor onset in PD (22). In PD, constipation is also frequently present along with slow gut motility and decreased fecal water content (23), dopaminergic deficits in neurons of the gut (24), and widespread aSyn pathology (synucleinopathy) in ENS neurons (25). Identifying treatments that can reduce synucleinopathy could benefit millions worldwide.

We assessed the neuroprotective potential of FTY720 (fingolimod, Gilenya), a sphingosine analog that is Food and Drug Administration-approved for multiple sclerosis (26). FTY720 is an oral drug that readily crosses the blood-brain barrier (27). It is rapidly phosphorylated by sphingosine kinase 2 to form FTY720-P, which signals through sphingosine 1 phosphate receptors that are expressed on neurons and glia of the CNS as well as PNS (28). The drugs' mode of action includes an ability of FTY720-P to block T-cell egress from lymph nodes, thus preventing T-cell entry to the brain (27). However, we and others also find that FTY720-mediated neuroprotection occurs in association with its ability to increase BDNF levels *in vitro* and *in vivo* (26, 29–36). This may be associated with the ability of FTY720 to stimulate signaling through Akt, ERK, and CREB, which increases BDNF expression; however, the mechanisms underlying the effects of FTY720 in the gut have not been defined.

Here we measured the impact of oral FTY720 on the gut of young and aging WT littermates and A53T aSyn Tg mice (37–40). Gut function was assessed behaviorally, and synucleinopathy was evaluated using immunohistochemistry and sequential extraction plus immunoblots. BDNF mRNA and protein were measured using quantitative PCR and immunoblot, respectively, as well as by measuring levels of miR206-3p.

## Results

### 

#### 

##### Early Onset Synucleinopathy in A53T Gut Reduces Tyrosine Hydroxylase Staining in ENS Catecholaminergic Neurons

**FIGURE 1.**
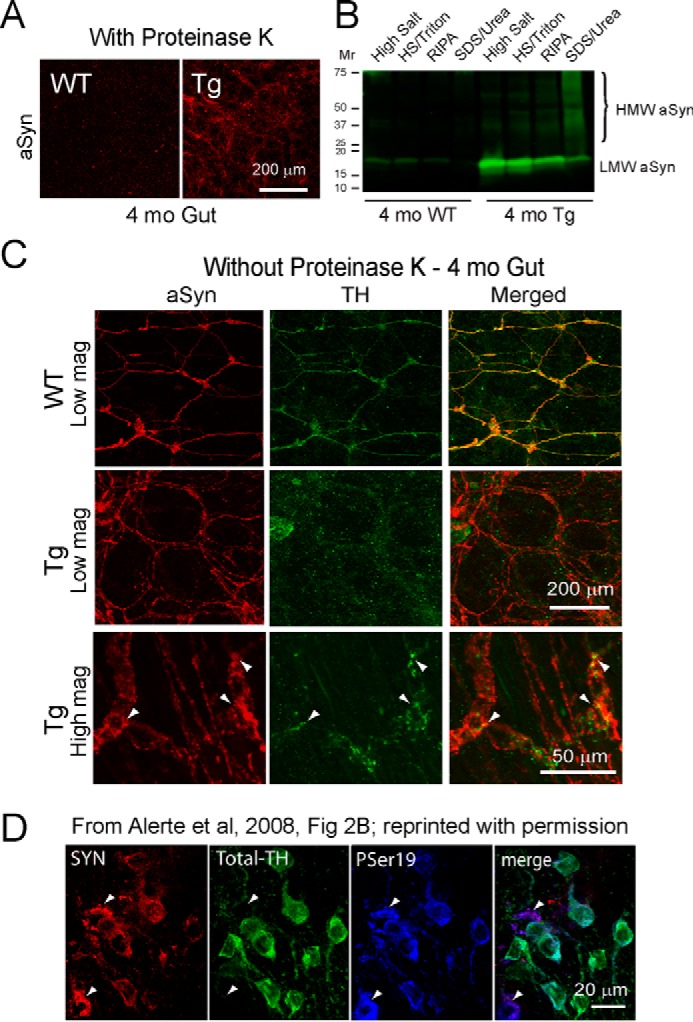
**Proteinase K-resistant aSyn aggregation in ENS and reduced TH signal in 4-month-old A53T Tg gut.** *A*, representative samples of gut tissue from 4-month-old A53T mice show that proteinase K eliminates most aSyn signal (*red*) from WT gut (*left*), whereas abundant proteinase K-resistant aSyn (*red*) remains in Tg gut (*right*), confirming early aSyn aggregation in Tg ENS. *B*, sequential extraction of gut tissue from 4-month-old littermates confirms soluble LMW aSyn in WT gut and abundant insoluble HMW aSyn in the Tg gut *SDS/urea lane. C*, gut tissues without proteinase K treatment show that WT littermates (*top row*) have a well colocalized aSyn (*red*) and TH (*green*) signal that appears *yellow* in the *merged image*. Although Tg gut (*middle row*) has a similar aSyn signal (*red*), there is much less TH (*green*) signal and reduced *merged yellow signal*. At high magnification, Tg gut (*bottom row*) shows that aSyn (*red*) colocalizes with TH (*green*) in ENS neurons (*arrowheads*), producing *yellow merged signal. D*, we previously found that CNS TH neurons with aggregated aSyn (*SYN*, *red*, *arrowheads*) have much less TH signal (*Total-TH*, *green*, *arrowheads*), although cells are intact, as confirmed using an antibody for phosphorylated TH serine 19 (*PSer19*, *blue*, *arrowheads*). Reduced total TH signal made cells look *purple* in the *merged image* (*merge*, *arrowheads*). *RIPA*, radioimmune precipitation assay buffer. Reprinted with permission from Elsevier, obtained via RightsLink Copyright Clearance Center (Alerte, T. N., Akinfolarin, A. A., Friedrich, E. E., Mader, S. A., Hong, C. S., and Perez, R. G. (2008) α-Synuclein aggregation alters tyrosine hydroxylase phosphorylation and immunoreactivity: lessons from viral transduction of knockout mice. *Neurosci. Lett.* **435,** 24–29).

Before testing FTY720 effects in A53T mice, we evaluated the onset of aSyn aggregation in Tg gut tissue pretreated with proteinase K, an enzyme that digests soluble proteins while leaving insoluble protein aggregates intact (41). In WT gut, proteinase K pretreatment eliminated most of the aSyn signal, suggesting that the aSyn was soluble (Fig. 1*A*, *left*). Abundant proteinase K-resistant aSyn immunoreactivity was still present in the gut of A53T Tg 4-month-old mice (Fig. 1*A*, *right*). For confirmation, we performed sequential extraction plus immunoblots of WT and Tg A53T gut tissue, which reconfirmed soluble aSyn in WT gut and abundant insoluble aSyn in Tg gut (Fig. 1*B*). As an additional measure, we also assessed 4-month gut tissue without proteinase K pretreatment to see whether aSyn colocalized with tyrosine hydroxylase (TH), a catecholaminergic neuronal marker. As expected, WT gut had well colocalized aSyn with TH in myenteric plexus neurons, which produced a *yellow* merged signal (Fig. 1*C*, *top row*). In contrast, 4-month Tg gut had strong aSyn signal but weak TH signal, producing little *yellow* signal in the *merged image* (Fig. 1*C*, *middle row*). The use of high magnification confocal microscopy allowed us to verify that TH and aSyn were colocalized in 4-month Tg gut catecholaminergic neurons, as demonstrated by *yellow* merged signal in ENS neurons (Fig. 1*C*, *bottom row*, *arrowheads*). This finding was reminiscent of our earlier discovery, reprinted here with permission, which shows that dopaminergic neurons harboring aggregated aSyn (Fig. 1*D*, *panel 1*, *red staining*, *arrowheads*) have almost no total TH signal (Fig. 1*D*, *panel 2*, *Total-TH*, note “missing *green* cells” at *arrowheads*) although cells were confirmed to be TH neurons using an antibody for TH phosphorylated on serine 19 (Fig. 1*D*, *PSer19*, *panel 3*, *blue staining*, *arrowheads*). The double labeling for aSyn (*red*) and TH PSer19 (*blue*) produced a *purple* merged signal (Fig. 1*D*, *panel 4*, *arrowheads*) (42). This finding reveals that both CNS and PNS TH^+^ neurons harboring aSyn aggregates may no longer be apparent if only stained for total TH.

##### FTY720 Significantly Improves Gut Function in A53T Tg Mice

Although WT littermate mice never developed synucleinopathy as occurs in Tg littermates (37, 40), the WT mice served as a control to measure the impact of FTY720 in mice that lack parkinsonian features. We first assessed fecal water content in WT littermates and Tg mice at 5 months of age, before beginning treatment. We found that at this age, WT and Tg mice had similar fecal water content (WT, 181.4 ± 6.59 mg; Tg, 178.2 ± 5.24 mg; *p* = 0.6175). WT and Tg mice then were given oral vehicle or FTY720 (0.5 mg/kg) twice weekly for 15 months. Importantly, mice never developed loose stools or diarrhea in response to FTY720 treatment.

**FIGURE 2.**
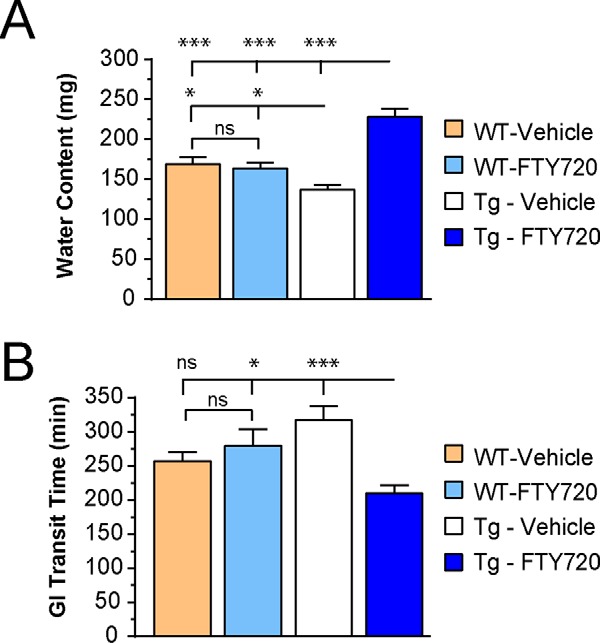
**Gut function is improved in Tg mice given oral FTY720, whereas FTY720 has little effect on WT mice.** *A*, fecal water content is similar in aged WT mice given vehicle or FTY720, whereas vehicle-treated Tg mice have a significant decrease in fecal water content, compatible with constipation, and FTY720-treated Tg mice have more water in the feces. *B*, WT mice had similar GI transit times when treated with vehicle or FTY720, whereas Tg mice given vehicle showed significant gut slowing, as compared with FTY720-treated Tg mice that had more rapid gut motility than any other group (*n* = 20 mice/treatment group); *ns*, not significant; *, *p* < 0.05; ***, *p* < 0.001. *Error bars*, S.E.

To measure constipation, we compared water content in feces and saw that within-group data were similar for all time points evaluated up to 15 months, so data were pooled for each condition (Fig. 2*A*). WT littermate mice given vehicle or FTY720 had similar water content. However, Tg mice treated with vehicle had significantly less water in feces than either WT mice or Tg mice treated with FTY720 (Fig. 2*A*, one-way ANOVA), suggesting that FTY720 may have decreased constipation in Tg mice.

As a more sensitive measure of gut function, we evaluated total gastrointestinal (GI) transit time in WT and Tg A53T mice treated with vehicle or FTY720. This involved measuring the time elapsed before mice eliminated the first red fecal pellet after carmine red gavage (as detailed under “Experimental Procedures”). Similar to water content, WT mice given vehicle or FTY720 had equivalent GI transit times. Tg mice given vehicle, however, had significantly slower GI transit time than WT mice or Tg given FTY720 (Fig. 2*B*, one-way ANOVA). These findings suggest that oral FTY720 significantly improved gut motility in Tg mice and also raised the possibility that FTY720 may have reduced gut synucleinopathy.

To determine whether gut length may have contributed to the above findings, we measured total gut length in age-matched WT and Tg littermate A53T mice (*n* = 6; WT, 46.25 ± 1.15 cm; Tg, 45.75 ± 0.75 cm; *p* = 0.73), which was not different. Because WT mice had no gut dysfunction up to 15 months, further comparisons were made using Tg mice that develop extensive synucleinopathy with age (40).

##### FTY720 Continues to Improve Gut Function in Old Tg Mice

**FIGURE 3.**
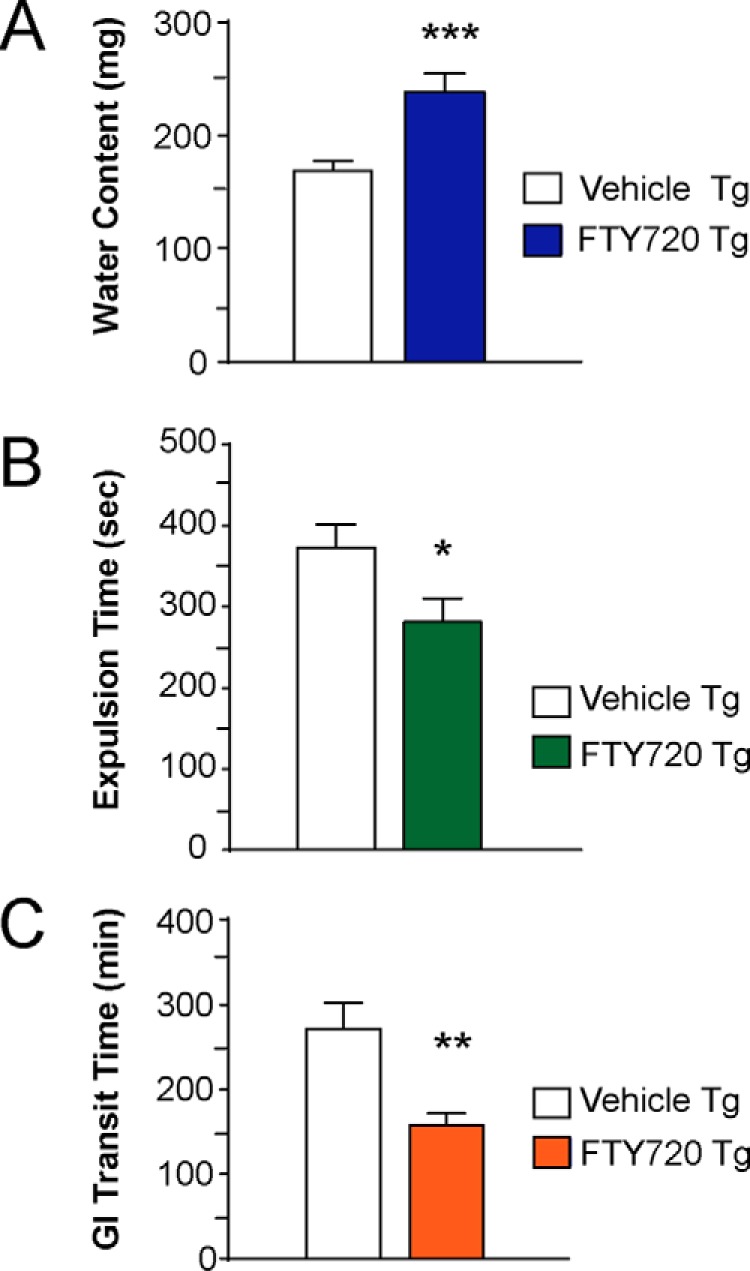
**Gut function is sustained in aged Tg mice on long term oral FTY720.** In Tg mice at 17–22 months (*A*), FTY720 significantly improves fecal water content. *B*, colonic motility, assessed using the bead expulsion test, shows improved colonic motility after FTY720. *C*, total GI transit time was also significantly better in FTY720-treated Tg mice as compared with vehicle-treated Tg mice (*n* = 8 mice/treatment group); *, *p* < 0.05; **, *p* < 0.01; ***, *p* < 0.001. *Error bars*, S.E.

To evaluate whether the response to FTY720 was sustainable, we measured water content, colonic motility, and total GI transit time in 17–22-month-old Tg mice (*n* = 8 mice/group). Significantly greater fecal water content was seen in Tg mice given FTY720 as compared with Tg mice treated with vehicle (Fig. 3*A*, *t* test, *p* < 0.001). We also assessed colonic motility, by measuring expulsion of a small glass bead that was gently inserted into the colon in Tg mice (detailed under “Experimental Procedures”). This confirmed that old Tg mice given long term FTY720 had significantly better colonic motility than Tg mice on vehicle (Fig. 3*B*, *t* test, *p* < 0.05). We also measured total GI transit time, which was significantly better in Tg mice on long term oral FTY720 as compared with Tg mice on vehicle (Fig. 3*C*, *t* test, *p* < 0.01). Collectively, these findings suggest that long term FTY720 was well tolerated and that mice continue to improve, even at advanced ages. At the end of behavioral experiments, gut tissues were collected and evaluated as described below.

##### FTY720 Reduces Synucleinopathy in Aging A53T Tg Gut

**FIGURE 4.**
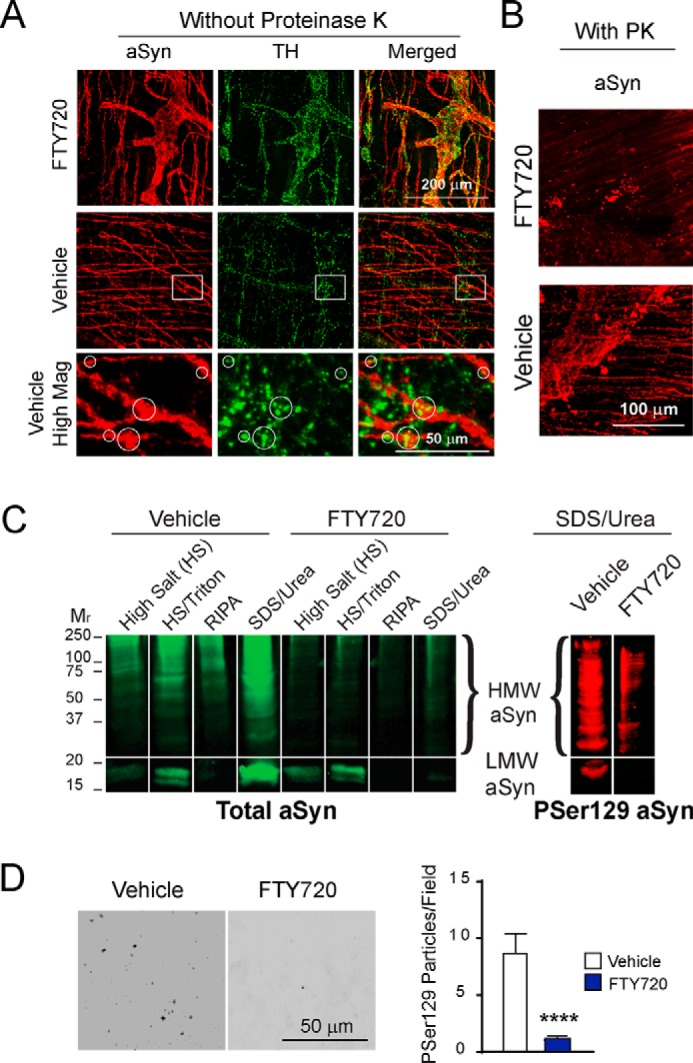
**Reduced aSyn pathology in A53T Tg mice after long term oral FTY720.** *A*, representative whole mounts of Tg colon that were not pretreated with proteinase K are from 17-month-old mice immunostained for aSyn (*red*) and TH (*green*). The *top row* shows low magnification images of FTY720-treated Tg colon stained for aSyn and TH, with abundant colocalized *yellow merged signal*. The *middle row* shows low magnification images of vehicle-treated Tg colon with much less aSyn/TH colocalization (*white boxed areas*). The *bottom row* shows high magnification images of *boxed areas* with *yellow merged signal* (*white circles*), confirming aSyn/TH colocalization in myenteric neurons. *B*, immunostaining of representative colons of 22-month-old Tg mice after 17 months of treatment with FTY720 or vehicle. Fewer proteinase K-resistant aSyn aggregates are present in FTY720-treated Tg colon (*top*), as compared with plentiful proteinase K-resistant aSyn aggregates in vehicle-treated Tg colon (*bottom*). *C*, sequential extraction of representative Tg colons from 21–22-month-old mice treated with vehicle or FTY720 run on a single gel displays abundant HMW aSyn in vehicle Tg mice and much less HMW aSyn in FTY720-treated Tg mice (total aSyn, sc-7011-R C20 antibody, *green signal*). Reprobing of the SDS/urea-insoluble sample for Ser(P)-129 (*PSer129*) aSyn shows abundant insoluble aSyn Ser(P)-129 in vehicle Tg gut as compared with FTY720-treated Tg gut (11A5 antibody, *red signal*). *D*, a representative 85-μm^2^ *black and white* microscopic field of Tg gut immunostained for aSyn Ser(P)-129. Samples were analyzed using ImageJ quantification in age-matched Tg mice for both conditions. The histogram shows quantification of gut Ser(P)-129 particles, which were significantly greater in number in vehicle than in FTY720 Tg gut (*n* = 8 mice/treatment group); ****, *p* < 0.0001. *RIPA*, radioimmune precipitation assay buffer. *Error bars*, S.E.

Gershon and colleagues (43–45) have shown that much of the body's dopamine lies in the gut, and our laboratory has long studied the relationship between aSyn and TH (40, 42, 46–49). Thus, we assessed colons of 17-month-old Tg mice for colocalization of aSyn and TH using immunohistochemistry. At low magnification, FTY720-treated Tg gut retained widespread colocalization, with abundant *yellow* merged aSyn and TH signal (Fig. 4*A*, *top row*), much like our findings in normal gut of 4-month-old WT littermate mice (Fig. 1*C*, *top row*). This contrasted with 17-month-old vehicle-treated Tg mice, which had much less aSyn/TH colocalization (Fig. 4*A*, *white boxes*, *middle row*). When images were enlarged, aSyn/TH colocalization is apparent in TH neurons of the myenteric plexus (Fig. 4*A*, *bottom row*). Reduced aSyn/TH colocalization in vehicle-treated Tg gut was similar to our earlier *in vivo* data for CNS neurons harboring aggregated aSyn (42), which had very little TH immunoreactivity (Fig. 1*D*, *arrowheads*). These findings suggested that FTY720 was able to reduce synucleinopathy in Tg gut.

We next assessed this in colons of vehicle- or FTY720-treated 22-month-old Tg mice that were immunostained for aSyn after pretreating with proteinase K. Fewer aSyn aggregates were present in FTY720-treated Tg gut (Fig. 4*B*, *top*), in contrast to robust aSyn aggregation in vehicle-treated Tg gut (Fig. 4*B*, *bottom*), much like our previous findings in Tg mice (40).

Further assessment of aSyn aggregation was done with sequential protein extraction of colons from vehicle- and FTY720-treated Tg mice on immunoblots probed for aSyn (Fig. 4*C*) as before (49). Ponceau staining of the immunoblot before aSyn probing confirmed equivalent protein in all lanes (not shown), similar to Fig. 6*B* (*right*). Vehicle Tg colon had more high molecular weight (HMW) aSyn as compared with FTY720 Tg colon, which had much less HMW aSyn, especially in the SDS/urea-insoluble samples (Fig. 4*C*, *green blot*). When we reprobed SDS/urea samples for aSyn phosphorylated on serine 129 (Ser(P)-129), a species that is abundant in Lewy bodies (50, 51), there was more Ser(P)-129 aSyn in vehicle as compared with FTY720 Tg mice (Fig. 4*C*, compare *left* and *right bands*, *red blot*). We also performed Ser(P)-129 aSyn immunostaining on Tg vehicle and Tg FTY720 gut for quantification, which showed significantly fewer Ser(P)-129 particles/unit area in FTY720-treated Tg mice (Fig. 4*D*; vehicle, 8.5 ± 2.2; FTY720, 1.0 ± 0.26; *t* test, *p* < 0.0001). This also suggests that long term FTY720 slows aSyn aggregation in neurons of aging Tg mice. We next explored potential mechanisms underlying the FTY720 effects.

##### FTY720 Increases Gut BDNF in Old A53T Tg Mice in Association with miR206-3p Down-regulation

**FIGURE 5.**
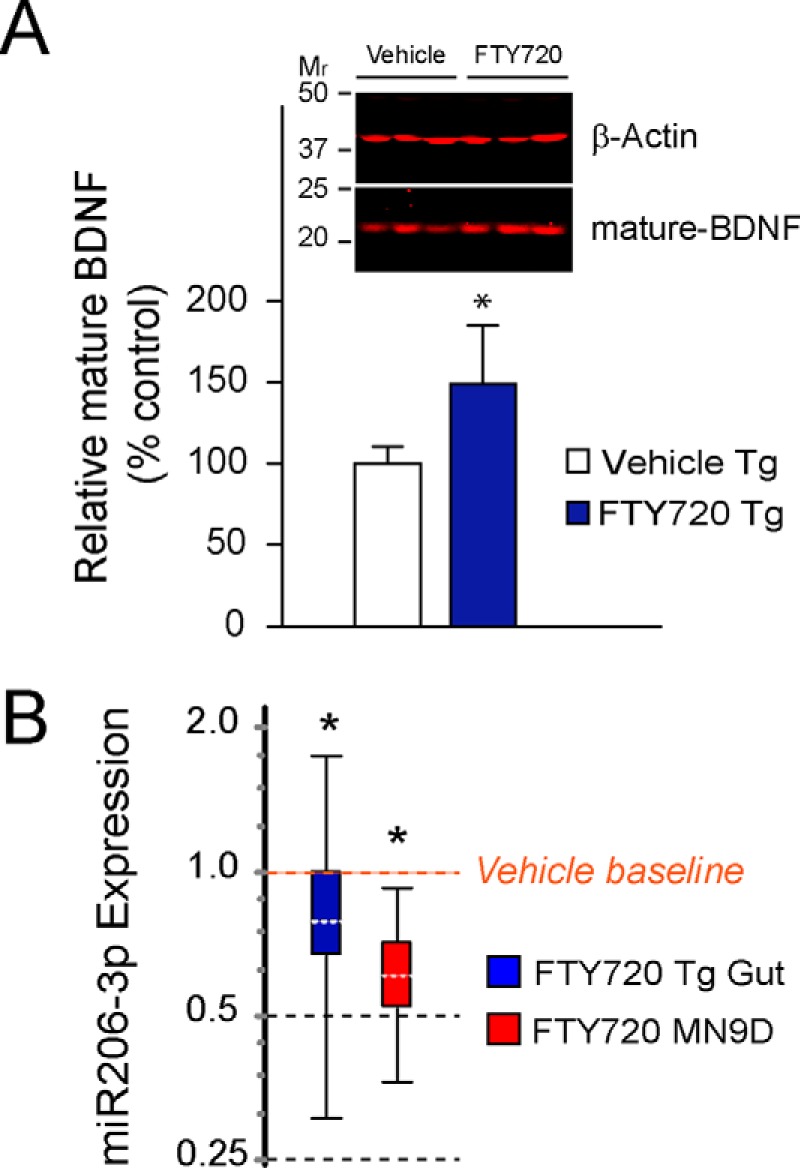
**FTY720 stimulates long term increases in BDNF protein in aging Tg mice in association with significantly lower levels of miR206–3p.** *A*, BDNF protein normalized to β-actin on immunoblots confirmed that BDNF was increased in colons of FTY720-treated 21-month-old mice. *B*, the expression of the regulatory microRNA, miR206–3p, was significantly lower in response to FTY720 treatment of aged Tg mice as compared with vehicle Tg mice. The decrease in miR206–3p was further validated in a control experiment with dopaminergic MN9D cells treated with 160 nm FTY720 for 24 h. (*n* = 8 mice/treatment group); *, *p* < 0.05; ***, *p* < 0.001. *Error bars*, S.E. for 5*A*, but not for REST 2009 analyses in 5*B*, as explained in Statistical Methods.

BDNF is well expressed in the gut (52), which contributes significantly to gut motility (53, 54). We and others have shown previously that FTY720 significantly increases BDNF expression *in vitro* and *in vivo* (26, 29–35). Using immunoblots, we evaluated BDNF protein levels in Tg gut after 17 months of vehicle or FTY720 in 22-month-old mice treated from 5 months of age. We saw significantly more mature BDNF in FTY720 Tg gut as compared with vehicle control (Fig. 5*A*, vehicle, 100 ± 5.8; FTY720, 141 ± 30.2; *t* test, *p* < 0.5). We were thus somewhat surprised to find that BDNF mRNA levels were similar in both treatment groups of Tg mice (*p* = 0.97, *t* test). To assess how this may have occurred, we next measured levels of regulatory miRNAs in gut.

It is established that miRNAs can regulate BDNF expression *in vivo* and *in vitro* (55); thus, we screened miRNAs in the colon of aged Tg mice given vehicle or FTY720. We noted a significant decrease in miR206-3p in FTY720-treated Tg gut as compared with vehicle (Fig. 5*B*, *blue box plot*, *p* = 0.045). As an additional control, we measured miR206-3p expression in MN9D dopaminergic neuronal cells after treating them with FTY720, which we previously found increases BDNF levels in these cells (26). Similarly to Tg gut, MN9D cells also significantly decreased miR206-3p in response to FTY720 treatment (Fig. 5*B*, *red box plot*, *p* = 0.034), suggesting a mechanism whereby FTY720 may have improved gut motility by increasing BDNF levels. To further test the role for BDNF in FTY720-associated changes, we performed the following *in vivo* experiments wherein we blocked the activity of BDNF Trk-B receptors.

##### Trk-B Receptor Inhibition Induces Constipation That Is Reversed by FTY720

**FIGURE 6.**
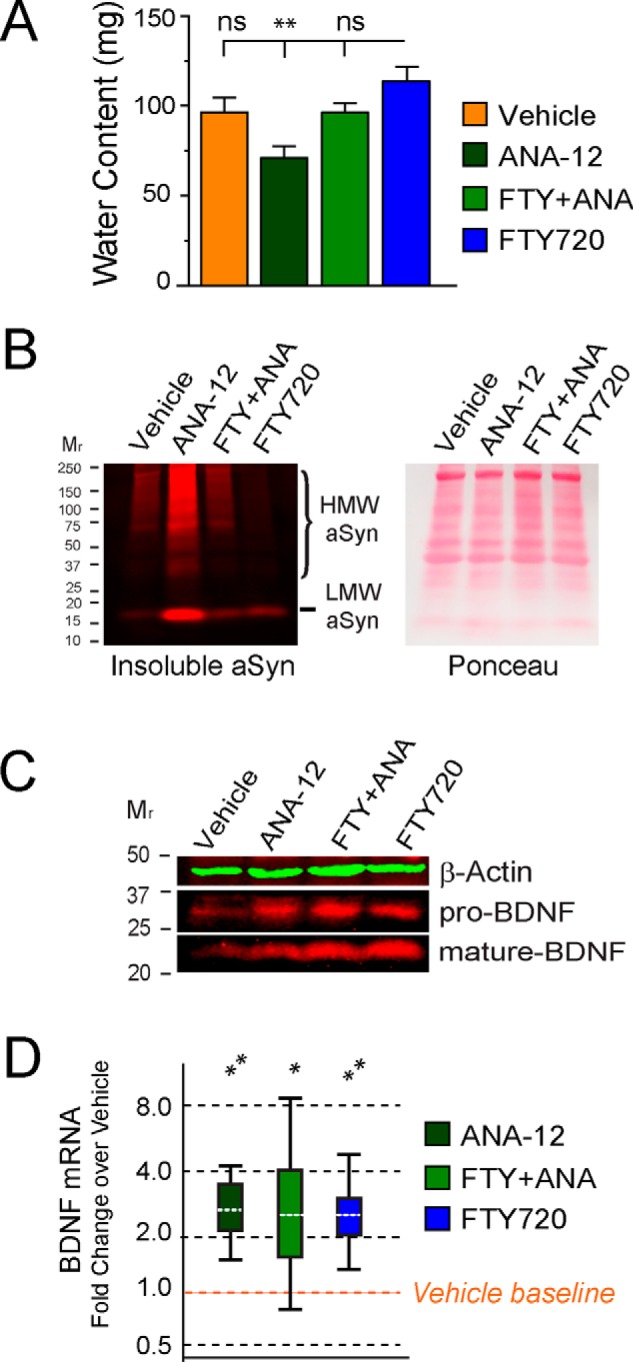
**ANA-12 inhibition of Trk-B BDNF signaling causes constipation and increases aSyn pathology as well as pro-BDNF and mature BDNF in 4-month-old A53T Tg gut.** *A*, young A53T mice were treated with vehicle alone, FTY720 alone (0.5 mg/kg), ANA-12 alone (0.5 mg/kg), or FTY720+ ANA-12 (0.5 mg/kg of each) for 3 months beginning at 1 month of age. Feces were collected to measure water content as described under “Experimental Procedures.” Only ANA-12-treated mice developed constipation in response to a loss of Trk-B signaling, whereas equivalent fecal water content was seen in all other conditions. *B*, *left*, SDS/urea-insoluble aSyn immunoblot of gut from mice treated with vehicle, ANA-12, FTY720 + ANA-12, and FTY720. The most abundant HMW aSyn is seen in the colon of ANA-12 Tg mice, some but less in vehicle and FTY720 + ANA-12 treatments, and very little insoluble aSyn in Tg colon after a 3-month treatment. All *lanes* had equal protein loading, as demonstrated in the Ponceau-stained blot on the *right. C*, immunoblots further reveal that both pro-BDNF and mature BDNF increased above baseline levels in vehicle Tg gut. β-Actin is the loading control. *D*, BDNF mRNA levels also increased above levels present in vehicle-treated Tg colon (*n* = 19 mice); **, *p* < 0.01; ***, *p* < 0.001. *Error bars*, S.E. for 6*A*, but not for REST 2009 analyses in 6*D*, as explained in Statistical Methods.

Using a TrkB receptor-specific antagonist, ANA-12 (56), given as described under “Experimental Procedures,” we performed the following experiment in littermate A53T mice (*n* = 19) treated from 1 to 4 months of age. Conditions included vehicle, FTY720, ANA-12, or FTY720 + ANA-12. Fecal water content within groups was similar at all times evaluated, so we combined data for assessments performed at 2, 3, and 4 months (Fig. 6). ANA-12 significantly reduced fecal water content as compared with FTY720 alone, while vehicle or FTY720 + ANA-12 mice were not significantly different from mice treated with FTY720 (Fig. 6*A*, one-way ANOVA, *p* < 0.01). This suggests that Trk-B-associated BDNF signaling may not be the sole factor underlying FTY720-mediated improvement in the gut of A53T Tg mice.

To assess this possibility, we collected colons to measure aSyn aggregation using sequential protein extraction. Ponceau staining confirmed equivalent protein loading (Fig. 6*B*, *right*). As can be appreciated in Fig. 6*B* (*left*), both HMW and low molecular weight (LMW) species of insoluble aSyn were seen in vehicle-treated Tg colon (Fig. 6*B*, *lane 1*), similar to what we previously saw in untreated 4-month-old Tg gut (Fig. 1*B*, *SDS/urea lane*). Curiously, HMW aSyn in ANA-12-treated Tg colon was markedly increased 14-fold above vehicle after a 3-month Trk-B blockade, suggesting that loss of BDNF signaling in the gut may accelerate ENS synucleinopathy. FTY720 + ANA-12-co-treated Tg mice had levels of HMW aSyn (Fig. 6*B*, *lane 3*) much like those of vehicle-treated mice (Fig. 6*B*, *lane 1*). In stark contrast, FTY720-treated Tg mice had almost no aggregated HMW aSyn present in the colon (Fig. 6*B*, *lane 4*).

We then measured BDNF protein and mRNA in colons of the 4-month-old mice that had been treated for 3 months with vehicle, FTY720, ANA-12, or FTY720 + ANA-12 and saw that both pro-BDNF and mature BDNF protein were increased on immunoblots (Fig. 6*C*). When normalized to β-actin, ANA-12 and FTY720 + ANA-12 treatments both increased pro-BDNF protein levels ∼2.5-fold, whereas FTY720 produced a larger 3.1-fold increase in pro-BDNF. Levels of BDNF mRNA were similarly increased above vehicle levels for mice given ANA-12, FTY720 + ANA-12, or FTY720 (∼2.4–2.5-fold) (Fig. 6*D*), which is quite different from our findings in old A53T mice that showed no change in BDNF mRNA yet had significantly more mature BDNF and a decrease in miRNA206-3p, which was associated with a parallel increase in BDNF protein. The young A53T mice in our ANA-12 studies showed no changes in miR206-3p in any treatment condition (not shown).

## Discussion

Synucleinopathy is present early in the gut of many PD patients, leading some to propose that aSyn pathology might spread in a prion-like manner from gut to brain (57, 58), a concept still debated (59). Nonetheless, utilizing early pre-motor PD symptoms, such as anosmia, anxiety, depression, or constipation, in combination with small biopsies to measure aSyn pathology (17–19, 21, 60) could offer hope to identify patients at early PD stages when neuroprotective therapies may prevent the loss of nigrostriatal dopaminergic neurons. To this end, we undertook a long term preclinical study to measure FTY720 (fingolimod/Gilenya) effects on neuronal aSyn pathology and gut function in aging A53T Tg synucleinopathy mice. In mice up to 15 months of age, we also assessed FTY720 impact in the gut of WT littermate mice. We also confirmed that gut length was similar in all mice.

Neuroprotective strategies are highly sought for PD because they may act to slow or halt disease progression, especially if initiated before an extensive loss of nigrostriatal dopaminergic neurons (61). It has long been appreciated that levels of BDNF are reduced in PD brain and that BDNF is a key neurotrophin that enhances the survival of nigral dopaminergic neurons (62–64). Thus, one strategy to reduce neurodegeneration has focused on BDNF (65–67). Moreover, individuals who are homozygous for a G196A single nucleotide polymorphism in BDNF have delayed PD onset by ∼5 years (68). BDNF therapeutic approaches have included infusion of BDNF itself as well as delivery of BDNF by cell and/or viral methodologies. Although these strategies may work in preclinical models, such methods may be problematic in the clinic (69). Thus, it is timely to identify new therapies that can up-regulate *endogenous* BDNF expression (70), as we demonstrate here for FTY720.

**FIGURE 7.**
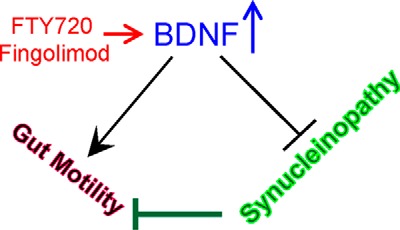
**Hypothetical model of FTY720-mediated stimulation of BDNF related effects on gut function and synucleinopathy.** Synucleinopathy in the ENS is hypothesized to contribute to poor gut motility. Oral FTY720/fingolimod stimulates the expression of gut BDNF, which improves gut motility and reduces ENS aSyn aggregation in young and old A53T Tg synucleinopathy mice. Blocking BDNF signaling also contributes to synucleinopathy. FTY720 may help to reverse this.

We tested the preclinical efficacy of long term FTY720 in aging A53T synucleinopathy mice that develop progressive aSyn pathology (40). Using this model allowed us to show for the first time that 1) long term FTY720 can be well tolerated and significantly improve gut function (Figs. 2 and 3); 2) FTY720 significantly reduces gut aSyn pathology even when given after the onset of synucleinopathy (Figs. 4 and 6); 3) FTY720 stimulates early and sustained up-regulation of BDNF (Figs. 5 and 6), which in young mice increased both pro-BDNF and mature BDNF and in old mice increased mature BDNF in association with reduced miR206-3p (Fig. 5); 4) blockade of Trk-B receptors in young A53T Tg mice significantly increased aSyn levels and aSyn aggregation in the gut; and 5) there is a huge loss of total TH immunoreactivity in neurons of the PNS containing aggregated aSyn (Figs. 1 and 4), similar to our prior findings in CNS dopaminergic neurons (42). Cumulatively, the data lead us to propose a model (Fig. 7) in which FTY720 stimulates BDNF expression, which improves gut motility and reduces gut synucleinopathy in the ENS of young (Fig. 6) and old synucleinopathy mice (Fig. 5).

Because both young and old Tg mice had improved gut function as well as reduced aSyn pathology after FTY720, the effects probably occur in response to mature BDNF, as demonstrated previously *in vitro* and *in vivo* (26, 29–36). It is intriguing that ANA-12 alone or in combination with FTY720 also stimulated an increase in BDNF protein and mRNA in mice, perhaps as an attempt to restore Trk-B signaling in the gut when signaling was blocked by ANA-12. Pro-BDNF, which acts through p75^NTR^ receptors, may also have contributed to normal fecal water content seen in A53T mice co-treated with FTY720 + ANA-12. It is known that pro-BDNF is more abundant in young mice, where it is secreted from neurons and signals through p75^NTR^ receptors, which contrasts with adult mice that normally have higher levels of mature BDNF (71), a finding corroborated here. It is accepted that p75^NTR^ receptor activity is not affected by ANA-12. However, pro-BDNF can be converted to mature BDNF, which may also have contributed to the increases in mature BDNF noted.

Additionally, there is evidence that brain BDNF levels can be increased by treating mice with the toxin MPTP (72). Moreover, paraquat or MPTP treatments increase aSyn protein levels and aSyn aggregation in the CNS of mice (73, 74), raising the possibility that ANA-12 may have induced oxidative stress in neurons in response to long term Trk-B receptor blockade. Finally, miRNAs are thought to play a role in age-associated neurodegeneration (75), and we saw changes in miR206-3p only in aged A53T Tg mice.

In sum, our translational studies of FTY720 (fingolimod/Gilenya) suggest that this widely used multiple sclerosis drug may have the potential to improve the quality of life for patients with PD and other synucleinopathies, such as multiple system atrophy (76, 77). Because the drug is already approved by the Food and Drug Administration, it could be rapidly tested for the ability to provide relief from the complications of synucleinopathy and related neurodegeneration.

## Experimental Procedures

### Mice

A53T aSyn (B6;C3-Tg-Prnp/SNCA*A53T/83Vle/J; Jackson Laboratories, Bar Harbor, ME) mice were used to generate our cohort of mice from A53T heterozygous breeders, which produced both WT and Tg mice. The Tg mice included heterozygous and homozygous offspring that overexpress one or two copies of A53T mutant human aSyn. Genotyping was performed as per Jackson Laboratories protocols. Mice were housed in barrier cages on ventilated racks in temperature and humidity-controlled rooms on 12-h light/dark cycles. Food and water were available *ad libitum*, except as noted below for particular experiments. Ethical treatment of animals followed AALAC, ARRIVE, and NIH guidelines performed on protocols approved by the Texas Tech University Health Sciences Center institutional animal care and use committee. A53T mice (*n* = 112) were randomly assigned to groups with data assessed by experimenters blinded to treatment conditions.

### Drug/Voluntary Oral Dosing

FTY720 (LC Laboratories, Woburn, MA) dissolved in 200 proof EtOH (vehicle) at a concentration of 29 mm was stored at −20 °C. Mice received FTY720 (0.5 mg/kg/mouse) or an equivalent amount of EtOH vehicle twice weekly by voluntary oral dosing using a modification of the jelly method (78) as described. Tablets were prepared from pulverized bacon softies (2.0 g; Bio-Serv, Flemington, NJ) and mouse chow (1.0 g; Harlan 8640 Teklad 22/5 rodent diet) mixed with 0.5 g of Splenda® in 2.0 ml of sterile MilliQ water to form a uniform paste. The paste was rolled to a uniform 0.2-cm thickness between sheets of plastic wrap. Tablets (0.5-cm diameter) were formed using a plastic transfer pipette cut 8 cm below the pipette neck (VWR, 414004-004, Westchester, PA) as a “cookie cutter.” Fresh tablets were prepared weekly. Mice in home cages were individually pretrained to eat an entire tablet in 1 min or less. Mice were food-restricted overnight to ensure ingestion of full tablets. Before each dose, mice were weighed, and tablets in 24-well tissue culture plates were inoculated with the correct volume of FTY720 or vehicle for each mouse.

For ANA-12 (Sigma-Aldrich), littermate mice received daily oral dosing of ANA-12 dissolved in DMSO (0.5 mg/kg/mouse) mixed with 10 μl of sesame oil and delivered by pipette. FTY720 (0.5 mg/kg/mouse), alone or in combination with ANA-12, was dissolved in DMSO and given twice weekly in sesame oil as described above. ANA-12 experiments included the following treatment groups: vehicle (*n* = 4), FTY720 (*n* = 4), ANA-12 (*n* = 3), or FTY720 + ANA-12 (*n* = 7).

### Behavioral Assessment

Mice were tested in clean quiet rooms in the Texas Tech University Health Sciences Center Laboratory Animal Resources Center facility a minimum of 2–3 times on separate occasions. We confirmed equivalent water intake in all mice.

#### 

##### Fecal Water Content

When food moves through the gut slowly, the colon absorbs more water, and consequently feces become dry and hard. Water content in feces was measured in 1–22-month-old mice, using methods described by Taylor *et al.* (79), with total stool collected in the afternoon from individual mice placed in clean cages for 1 h. Feces were immediately transferred to 1.5-ml Eppendorf tubes that were labeled, capped, and then weighed. Tubes were opened to allow desiccation of the contents on a heat block set to 65 °C overnight. Tubes were weighed again, and water content was calculated by computing the difference between wet and dry weight.

##### Colonic Motility

Colonic motility was measured in 15–22-month-old Tg mice using the bead expulsion test (80). Briefly, a glass bead (3 mm; Sigma-Aldrich, Z143928-1EA) was gently pushed 2.0 cm into the colon using the smooth end of a plastic inoculating loop (Nunc, 253287). The total time from bead insertion to bead ejection was recorded for each mouse.

##### Whole Gut Transit Time

Whole gut transit time was performed in 15–22-month-old Tg mice essentially as described by Kuo *et al.* (80). Briefly, transit time was assessed in mice after oral gavage of a 0.2-ml volume of 6% (w/v) carmine red dye in 0.5% methylcellulose (Sigma-Aldrich). Postgavage, the mice were observed for up to 9 h until the time of excretion of the first red stool, which was recorded for each mouse. Mice that had not passed red stool by 9 h were scored as >9 h.

### Tissue Collection and Preparation

Mice were euthanized by CO_2_ inhalation followed by decapitation. The gut was flushed of fecal contents, bisected along the longitudinal axis, and divided into samples for immunohistochemistry, biochemistry, and molecular biology. For immunohistochemistry, gut was pinned flat in Sylgard-coated Petri dishes with the lumen facing up, and tissue was fixed for 2–18 h in 4% formaldehyde/sucrose and then washed 3 times in PBS. For gut whole mounts, intestinal segments were dissected and trimmed into 1.5-cm cylinders and then fixed and processed using a modification of the method of Li *et al.* (81). For longitudinal muscle of the myenteric plexus, the villi and fatty gut tissues were gently scraped away under a dissecting microscope as described previously (40). Tissue for protein extraction was rapidly frozen on dry ice and then stored at −80 °C until use. Tissues for RNA extraction were preserved in RNALater solution (AM7021, Ambion, Thermo Fisher) as per the manufacturer's instructions. Before RNA extraction, tissues were frozen on liquid nitrogen and then crushed, with miRNA extraction performed immediately as described below. To measure gut length in 15-month-old littermates (*n* = 6), whole gut was cut from the base of the stomach to the anus. The entire gut was then carefully extended along a ruler, and the length in cm was documented.

### Sequential Protein Extraction

Protein extraction from colon was performed using the method of Waxman and Giasson (82) as detailed by Wu *et al.* (49). This method does not isolate particular cellular or subcellular fractions but rather isolates soluble and insoluble proteins using a series of buffers and re-extraction of pellets performed using ultracentrifugation.

### Immunohistochemistry

#### 

##### Gut

Gut tissues were immunolabeled for confocal microscopy using established methods and the Olympus FluoView 1000 system as before (40).

##### Antibodies and/Proteinase K Treatment

Immunohistochemistry was done on free floating sections with antibodies for aSyn (C20, sc-7011-R, Santa Cruz Biotechnology, Inc.) and phosphorylated aSyn Ser(P)-129 (SAB4503996, Sigma/Aldrich). For visualization of protein aggregates, tissue was first treated with proteinase K to digest soluble proteins as described previously (42). After washing and blocking tissues, gut sections were incubated for 18–24 h at 4 °C with aSyn antibody (1:100; sc-7011-R, Santa Cruz Biotechnology) followed by washes and incubation in goat anti-rabbit Alexa-546 (A-11035, Invitrogen). Some aSyn-labeled tissues were also labeled with an antibody for TH (chicken anti-TH, 1:200–1:250; Aves Laboratories, Tigard, OR).

### Quantification of Gut aSyn Ser(P)-129

Confocal *z*-stack images of 5 random fields/colon were collected from tissues immunostained for aSyn Ser(P)-129 in a subset of mice treated with vehicle or FTY720 for at least a year (*n* = 10). Particles >2 μm in diameter were counted on 85-μm^2^ fields of duodenal tissue evaluated using ImageJ (83) followed by manual analyses.

### Immunoblots

Proteins (50 μg) were separated by SDS-PAGE, transferred to nitrocellulose, blocked, and then incubated in primary antibody overnight at 4 °C. Antibodies included total aSyn (sc-7011-R, Santa Cruz Biotechnology), aSyn Ser(P)-129 (11A5, gift of Elan Pharmaceuticals), BDNF (N20, sc-546, Santa Cruz Biotechnology), and β-actin (4970, Cell Signaling, Danvers, MA). All blots were imaged and quantified using the LI-COR Odyssey and/or ImageQuant software as described previously (40, 84).

### Gene Expression

Total mRNA and miRNA were extracted from mouse gut tissue and MN9D cells using the miRNeasy minikit (Qiagen, catalog no. 217004) and RNase-free DNase kit (Qiagen, catalog no. 79254) according to the manufacturer. Retrotranscription of mRNAs and mature miRNAs was performed using the High Capacity RNA-to-cDNA kit (Applied Biosystems, catalog no. 4387406) and miScript II RT Kit (Qiagen, catalog no. 218160), respectively, as per the manufacturer's instructions. RNA concentration and purity was assessed by NanoDrop 2000 spectrophotometry (Fisher). RNA integrity and genomic DNA contamination were assessed using 28S/18S band ratios from RNA “bleach” gels exactly as described (85). Amplification was measured using quantitative real-time PCR in a RealPlex Mastercycler 2 instrument (Eppendorf Inc., Westbury, NY). Relative expression of mRNAs was measured using Taqman probe assays (Life Technologies, Inc.) for BDNF (catalog no. Mm04230607_s1) with GAPDH (catalog no. Mm99999915_g1) as an internal expression control. Relative expression of miR206-3p was measured using the mature miR206-3p miScript primer assay (catalog no. MS00001869) with miScript SNORD72 (catalog no. MS00033719) and SNORD95 (catalog no. MS00033726) primer assays as internal expression controls and miScript miRTC (catalog no. MS00000001) primer assays as internal retrotranscription controls (Qiagen).

### MN9D Cells

Cells were grown using established methods (46, 48, 84, 86) and treated with vehicle or 160 nm FTY720 for 24 h as described previously (26). Afterward, cells were collected and processed for microRNA assessment as described above.

### Statistics

Independent-sample *t* tests, ANOVA by Kruskal-Wallis, and Dunn's multiple comparisons tests were performed using Prism 6 (GraphPad Software Inc., San Diego, CA), with significance set to *p* < 0.05. BDNF mRNA and miR206-3p expression were calculated using the comparative *Ct* method (2^−ΔΔ^*^Ct^*) and relative expression software tool (REST) that is available online (87). Molecular and biochemical assays from 2–3 independent experiments were performed in duplicate or triplicate. Data represent means ± S.E., except for miR206-3p and BDNF whisker box plots generated using REST 2009 software, which demonstrate the median (*white dashed line* in *boxes*), interquartile ranges 1 and 3 (*top* and *bottom edges* of *boxes*), and maximum and minimum expression values (*top* and *bottom whiskers*).

## Author Contributions

G. V.-M. and S. J. D. did mouse breeding and behavior studies. G. V.-M. and B. Y. did genotyping and data analyses. J. V.-M. did protein chemistry, sequential extractions, and prepare figures. D. M. and C. G.-T. did immunohistochemistry, confocal microscopy, and data analyses and helped make figures. I. S.-U. identified and standardized the methods used by B. Y. and N. T. G. for mRNA and miRNA experiment, and helped write the manuscript. R. G. P. conceived of the project, made final figures, and wrote most of the paper.

## Footnotes

2 The abbreviations used are:

aSyn: α-synuclein
BDNF: brain-derived neurotrophic factor
ENS: enteric nervous system
GI: gastrointestinal
HMW: high molecular weight
LMW: low molecular weight
miR206-3p: microRNA 206-3p
miRNA: microRNA
MPTP: 1-methyl-4-phenyl-1,2,3,6-tetrahydropyridine
PD: Parkinson's disease
PNS: peripheral nervous system
Tg: A53T transgenic mice
TH: tyrosine hydroxylase
ANOVA: analysis of variance.

## References

[1] Ahn M., Kim S., Kang M., Ryu Y., and Kim T. D. (2006) Chaperone-like activities of α-synuclein: α-synuclein assists enzyme activities of esterases. Biochem. Biophys. Res. Commun. 346, 1142–1149 16796993 10.1016/j.bbrc.2006.05.213

[2] Kim T. D., Paik S. R., Yang C. H., and Kim J. (2000) Structural changes in α-synuclein affect its chaperone-like activity *in vitro*. Protein Sci. 9, 2489–2496 11206070 10.1110/ps.9.12.2489 PMC2144529

[3] Maroteaux L., Campanelli J. T., and Scheller R. H. (1988) Synuclein: a neuron-specific protein localized to the nucleus and presynaptic nerve terminal. J. Neurosci. 8, 2804–2815 3411354 10.1523/JNEUROSCI.08-08-02804.1988 PMC6569395

[4] Braak H., and Braak E. (2000) Pathoanatomy of Parkinson's disease. J. Neurol. 247, II3–II10 10991663 10.1007/PL00007758

[5] Goedert M., Spillantini M. G., Del Tredici K., and Braak H. (2013) 100 years of Lewy pathology. Nat. Rev. Neurol. 9, 13–24 23183883 10.1038/nrneurol.2012.242

[6] Krüger R., Kuhn W., Müller T., Woitalla D., Graeber M., Kösel S., Przuntek H., Epplen J. T., Schöls L., and Riess O. (1998) Ala30Pro mutation in the gene encoding α-synuclein in Parkinson's disease. Nat. Genet. 18, 106–108 9462735 10.1038/ng0298-106

[7] Cronin K. D., Ge D., Manninger P., Linnertz C., Rossoshek A., Orrison B. M., Bernard D. J., El-Agnaf O. M., Schlossmacher M. G., Nussbaum R. L., and Chiba-Falek O. (2009) Expansion of the Parkinson disease-associated SNCA-Rep1 allele upregulates human α-synuclein in transgenic mouse brain. Hum. Mol. Genet. 18, 3274–3285 19498036 10.1093/hmg/ddp265 PMC2722989

[8] Pasanen P., Myllykangas L., Siitonen M., Raunio A., Kaakkola S., Lyytinen J., Tienari P. J., P−oyh−onen M., and Paetau A. (2014) Novel α-synuclein mutation A53E associated with atypical multiple system atrophy and Parkinson's disease-type pathology. Neurobiol. Aging 35, 2180.e1–2180.e5 24746362 10.1016/j.neurobiolaging.2014.03.024

[9] Appel-Cresswell S., Vilarino-Guell C., Encarnacion M., Sherman H., Yu I., Shah B., Weir D., Thompson C., Szu-Tu C., Trinh J., Aasly J. O., Rajput A., Rajput A. H., Jon Stoessl A., and Farrer M. J. (2013) α-Synuclein p.H50Q, a novel pathogenic mutation for Parkinson's disease. Movement Disord. 28, 811–813 23457019 10.1002/mds.25421

[10] Lesage S., Anheim M., Letournel F., Bousset L., Honoré A., Rozas N., Pieri L., Madiona K., Dürr A., Melki R., Verny C., Brice A., and French Parkinson's Disease Genetics Study Group (2013) G51D α-synuclein mutation causes a novel Parkinsonian-pyramidal syndrome. Ann. Neurol. 73, 459–471 23526723 10.1002/ana.23894

[11] Singleton A. B., Farrer M., Johnson J., Singleton A., Hague S., Kachergus J., Hulihan M., Peuralinna T., Dutra A., Nussbaum R., Lincoln S., Crawley A., Hanson M., Maraganore D., Adler C., et al (2003) alpha-Synuclein locus triplication causes Parkinson's disease. Science 302, 841 14593171 10.1126/science.1090278

[12] Chartier-Harlin M.-C., Kachergus J., Roumier C., Mouroux V., Douay X., Lincoln S., Levecque C., Larvor L., Andrieux J., Hulihan M., Waucquier N., Defebvre L., Amouyel P., Farrer M., and Destée A. (2004) α-Synuclein locus duplication as a cause of familial Parkinson's disease. Lancet 364, 1167–1169 15451224 10.1016/S0140-6736(04)17103-1

[13] Polymeropoulos M. H., Lavedan C., Leroy E., Ide S. E., Dehejia A., Dutra A., Pike B., Root H., Rubenstein J., Boyer R., Stenroos E. S., Chandrasekharappa S., Athanassiadou A., Papapetropoulos T., Johnson W. G., et al (1997) Mutation in the alpha-synuclein gene identified in families with Parkinson's disease. Science 276, 2045–2047 9197268 10.1126/science.276.5321.2045

[14] Zarranz J. J., Alegre J., Gómez-Esteban J., Lezcano E., Ros R., Ampuero I., Vidal L., Hoenicka J., Rodriguez O., Atarés B., Llorens V., Gomez Tortosa E., del Ser T., Muñoz D. G., and de Yebenes J. G. (2004) The new mutation, E46K, of α-synuclein causes parkinson and Lewy body dementia. Ann. Neurol. 55, 164–173 14755719 10.1002/ana.10795

[15] Baba M., Nakajo S., Tu P. H., Tomita T., Nakaya K., Lee V. M., Trojanowski J. Q., and Iwatsubo T. (1998) Aggregation of α-synuclein in Lewy bodies of sporadic Parkinson's disease and dementia with Lewy bodies. Am. J. Pathol. 152, 879–884 9546347 PMC1858234

[16] Bernheimer H., Birkmayer W., Hornykiewicz O., Jellinger K., and Seitelberger F. (1973) Brain dopamine and the syndromes of Parkinson and Huntington: clinical, morphological and neurochemical correlations. J. Neurol. Sci. 20, 415–455 4272516 10.1016/0022-510x(73)90175-5

[17] Salawu F. K., Danburam A., and Olokoba A. B. (2010) Non-motor symptoms of Parkinson's disease: diagnosis and management. Niger. J. Med. 19, 126–131 20642073 10.4314/njm.v19i2.56496

[18] Pellicano C., Benincasa D., Pisani V., Buttarelli F. R., Giovannelli M., and Pontieri F. E. (2007) Prodromal non-motor symptoms of Parkinson's disease. Neuropsychiatr. Dis. Treat. 3, 145–152 19300544 10.2147/nedt.2007.3.1.145 PMC2654529

[19] Wolters E. (2009) Non-motor extranigral signs and symptoms in Parkinson's disease. Parkinsonism Relat. Disord. 15, S6–S12 20083010 10.1016/S1353-8020(09)70770-9

[20] Shannon K. M., Keshavarzian A., Dodiya H. B., Jakate S., and Kordower J. H. (2012) Is α-synuclein in the colon a biomarker for premotor Parkinson's disease? Evidence from 3 cases. Movement Disord. 27, 716–719 22550057 10.1002/mds.25020

[21] Shannon K. M., Keshavarzian A., Mutlu E., Dodiya H. B., Daian D., Jaglin J. A., and Kordower J. H. (2012) α-Synuclein in colonic submucosa in early untreated Parkinson's disease. Movement Disord. 27, 709–715 21766334 10.1002/mds.23838

[22] Savica R., Carlin J. M., Grossardt B. R., Bower J. H., Ahlskog J. E., Maraganore D. M., Bharucha A. E., and Rocca W. A. (2009) Medical records documentation of constipation preceding Parkinson disease: a case-control study. Neurology 73, 1752–1758 19933976 10.1212/WNL.0b013e3181c34af5 PMC2788809

[23] Ashraf W., Pfeiffer R. F., Park F., Lof J., and Quigley E. M. (1997) Constipation in Parkinson's disease: objective assessment and response to psyllium. Movement Disord. 12, 946–951 9399219 10.1002/mds.870120617

[24] Singaram C., Ashraf W., Gaumnitz E. A., Torbey C., Sengupta A., Pfeiffer R., and Quigley E. M. (1995) Dopaminergic defect of enteric nervous system in Parkinson's disease patients with chronic constipation. Lancet 346, 861–864 7564669 10.1016/s0140-6736(95)92707-7

[25] Braak H., de Vos R. A., Bohl J., and Del Tredici K. (2006) Gastric α-synuclein immunoreactive inclusions in Meissner's and Auerbach's plexuses in cases staged for Parkinson's disease-related brain pathology. Neurosci. Lett. 396, 67–72 16330147 10.1016/j.neulet.2005.11.012

[26] Vargas-Medrano J., Krishnamachari S., Villanueva E., Godfrey W. H., Lou H., Chinnasamy R., Arterburn J. B., and Perez R. G. (2014) Novel FTY720-based compounds stimulate neurotrophin expression and phosphatase activity in dopaminergic cells. ACS Med. Chem. Lett. 5, 782–786 25050165 10.1021/ml500128g PMC4094248

[27] Brinkmann V., Billich A., Baumruker T., Heining P., Schmouder R., Francis G., Aradhye S., and Burtin P. (2010) Fingolimod (FTY720): discovery and development of an oral drug to treat multiple sclerosis. Nat. Rev. Drug Discov. 9, 883–897 21031003 10.1038/nrd3248

[28] Nishimura H., Akiyama T., Irei I., Hamazaki S., and Sadahira Y. (2010) Cellular localization of sphingosine-1-phosphate receptor 1 expression in the human central nervous system. J. Histochem. Cytochem. 58, 847–856 20566754 10.1369/jhc.2010.956409 PMC2924800

[29] Deogracias R., Yazdani M., Dekkers M. P., Guy J., Ionescu M. C., Vogt K. E., and Barde Y. A. (2012) Fingolimod, a sphingosine-1 phosphate receptor modulator, increases BDNF levels and improves symptoms of a mouse model of Rett syndrome. Proc. Natl. Acad. Sci. U.S.A. 109, 14230–14235 22891354 10.1073/pnas.1206093109 PMC3435172

[30] Di Menna L., Molinaro G., Di Nuzzo L., Riozzi B., Zappulla C., Pozzilli C., Turrini R., Caraci F., Copani A., Battaglia G., Nicoletti F., and Bruno V. (2013) Fingolimod protects cultured cortical neurons against excitotoxic death. Pharmacol. Res. 67, 1–9 23073075 10.1016/j.phrs.2012.10.004

[31] Doi Y., Takeuchi H., Horiuchi H., Hanyu T., Kawanokuchi J., Jin S., Parajuli B., Sonobe Y., Mizuno T., and Suzumura A. (2013) Fingolimod phosphate attenuates oligomeric amyloid β-induced neurotoxicity via increased brain-derived neurotrophic factor expression in neurons. PLoS One 8, e61988 23593505 10.1371/journal.pone.0061988 PMC3625222

[32] Di Pardo A., Amico E., Favellato M., Castrataro R., Fucile S., Squitieri F., and Maglione V. (2014) FTY720 (fingolimod) is a neuroprotective and disease-modifying agent in cellular and mouse models of Huntington disease. Hum. Mol. Genet. 23, 2251–2265 24301680 10.1093/hmg/ddt615

[33] Fukumoto K., Mizoguchi H., Takeuchi H., Horiuchi H., Kawanokuchi J., Jin S., Mizuno T., and Suzumura A. (2014) Fingolimod increases brain-derived neurotrophic factor levels and ameliorates amyloid β-induced memory impairment. Behav. Brain Res. 268, 88–93 24713151 10.1016/j.bbr.2014.03.046

[34] Cipriani R., Chara J. C., Rodríguez-Antigüedad A., and Matute C. (2015) FTY720 attenuates excitotoxicity and neuroinflammation. J. Neuroinflammation 12, 86 25953296 10.1186/s12974-015-0308-6 PMC4429813

[35] Hait N. C., Wise L. E., Allegood J. C., O'Brien M., Avni D., Reeves T. M., Knapp P. E., Lu J., Luo C., Miles M. F., Milstien S., Lichtman A. H., and Spiegel S. (2014) Active, phosphorylated fingolimod inhibits histone deacetylases and facilitates fear extinction memory. Nat. Neurosci. 17, 971–980 24859201 10.1038/nn.3728 PMC4256678

[36] Miguez A., García-Díaz Barriga G., Brito V., Straccia M., Giralt A., Ginés S., Canals J. M., and Alberch J. (2015) Fingolimod (FTY720) enhances hippocampal synaptic plasticity and memory in Huntington's disease by preventing p75NTR up-regulation and astrocyte-mediated inflammation. Hum. Mol. Genet. 24, 4958–4970 26063761 10.1093/hmg/ddv218

[37] Giasson B. I., Duda J. E., Quinn S. M., Zhang B., Trojanowski J. Q., and Lee V. M. (2002) Neuronal α-synucleinopathy with severe movement disorder in mice expressing A53T human α-synuclein. Neuron 34, 521–533 12062037 10.1016/s0896-6273(02)00682-7

[38] Norris E. H., Uryu K., Leight S., Giasson B. I., Trojanowski J. Q., and Lee V. M. (2007) Pesticide exposure exacerbates α-synucleinopathy in an A53T transgenic mouse model. Am. J. Pathol. 170, 658–666 17255333 10.2353/ajpath.2007.060359 PMC1851868

[39] Gao H. M., Kotzbauer P. T., Uryu K., Leight S., Trojanowski J. Q., and Lee V. M. (2008) Neuroinflammation and oxidation/nitration of α-synuclein linked to dopaminergic neurodegeneration. J. Neurosci. 28, 7687–7698 18650345 10.1523/JNEUROSCI.0143-07.2008 PMC2702093

[40] Farrell K. F., Krishnamachari S., Villanueva E., Lou H., Alerte T. N., Peet E., Drolet R. E., and Perez R. G. (2014) Non-motor parkinsonian pathology in aging A53T α-synuclein mice is associated with progressive synucleinopathy and altered enzymatic function. J. Neurochem. 128, 536–546 24117685 10.1111/jnc.12481 PMC4283050

[41] Neumann M., Kahle P. J., Giasson B. I., Ozmen L., Borroni E., Spooren W., Müller V., Odoy S., Fujiwara H., Hasegawa M., Iwatsubo T., Trojanowski J. Q., Kretzschmar H. A., and Haass C. (2002) Misfolded proteinase K-resistant hyperphosphorylated α-synuclein in aged transgenic mice with locomotor deterioration and in human α-synucleinopathies. J. Clin. Invest. 110, 1429–1439 12438441 10.1172/JCI15777 PMC151810

[42] Alerte T. N., Akinfolarin A. A., Friedrich E. E., Mader S. A., Hong C. S., and Perez R. G. (2008) α-Synuclein aggregation alters tyrosine hydroxylase phosphorylation and immunoreactivity: lessons from viral transduction of knockout mice. Neurosci. Lett. 435, 24–29 18314273 10.1016/j.neulet.2008.02.014 PMC2440662

[43] Li Z., Chalazonitis A., Huang Y. Y., Mann J. J., Margolis K. G., Yang Q. M., Kim D. O., Cǒté F., Mallet J., and Gershon M. D. (2011) Essential roles of enteric neuronal serotonin in gastrointestinal motility and the development/survival of enteric dopaminergic neurons. J. Neurosci. 31, 8998–9009 21677183 10.1523/JNEUROSCI.6684-10.2011 PMC4442094

[44] Li Z. S., Pham T. D., Tamir H., Chen J. J., and Gershon M. D. (2004) Enteric dopaminergic neurons: definition, developmental lineage, and effects of extrinsic denervation. J. Neurosci. 24, 1330–1339 14960604 10.1523/JNEUROSCI.3982-03.2004 PMC6730344

[45] Li Z. S., Schmauss C., Cuenca A., Ratcliffe E., and Gershon M. D. (2006) Physiological modulation of intestinal motility by enteric dopaminergic neurons and the D2 receptor: analysis of dopamine receptor expression, location, development, and function in wild-type and knock-out mice. J. Neurosci. 26, 2798–2807 16525059 10.1523/JNEUROSCI.4720-05.2006 PMC6675162

[46] Peng X., Peng X. M., Tehranian R., Dietrich P., Stefanis L., and Perez R. G. (2005) α-Synuclein activation of protein phosphatase 2A reduces tyrosine hydroxylase phosphorylation in dopaminergic cells. J. Cell Sci. 118, 3523–3530 16030137 10.1242/jcs.02481

[47] Perez R. G., and Hastings T. G. (2004) Could a loss of α-synuclein function put dopaminergic neurons at risk? J. Neurochem. 89, 1318–1324 15189334 10.1111/j.1471-4159.2004.02423.x

[48] Perez R. G., Waymire J. C., Lin E., Liu J. J., Guo F., and Zigmond M. J. (2002) A role for α-synuclein in the regulation of dopamine biosynthesis. J. Neurosci. 22, 3090–3099 11943812 10.1523/JNEUROSCI.22-08-03090.2002 PMC6757524

[49] Wu J., Lou H., Alerte T. N., Stachowski E. K., Chen J., Singleton A. B., Hamilton R. L., and Perez R. G. (2012) Lewy-like aggregation of α-synuclein reduces protein phosphatase 2A activity *in vitro* and *in vivo*. Neuroscience 207, 288–297 22326202 10.1016/j.neuroscience.2012.01.028 PMC3570090

[50] Iwatsubo T. (2003) Aggregation of α-synuclein in the pathogenesis of Parkinson's disease. J. Neurol. 250, III11–III14 14579119 10.1007/s00415-003-1303-x

[51] Anderson J. P., Walker D. E., Goldstein J. M., de Laat R., Banducci K., Caccavello R. J., Barbour R., Huang J., Kling K., Lee M., Diep L., Keim P. S., Shen X., Chataway T., Schlossmacher M. G., Seubert P., Schenk D., Sinha S., Gai W. P., and Chilcote T. J. (2006) Phosphorylation of Ser-129 is the dominant pathological modification of α-synuclein in familial and sporadic Lewy body disease. J. Biol. Chem. 281, 29739–29752 16847063 10.1074/jbc.M600933200

[52] Lommatzsch M., Braun A., Mannsfeldt A., Botchkarev V. A., Botchkareva N. V., Paus R., Fischer A., Lewin G. R., and Renz H. (1999) Abundant production of brain-derived neurotrophic factor by adult visceral epithelia. Implications for paracrine and target-derived neurotrophic functions. Am. J. Pathol. 155, 1183–1193 10514401 10.1016/S0002-9440(10)65221-2 PMC1867012

[53] Chen F., Yu Y., Wang P., Dong Y., Wang T., Zuo X., and Li Y. (2014) Brain-derived neurotrophic factor accelerates gut motility in slow-transit constipation. Acta Physiol. 212, 226–238 10.1111/apha.12374 25164090

[54] Coulie B., Szarka L. A., Camilleri M., Burton D. D., McKinzie S., Stambler N., and Cedarbaum J. M. (2000) Recombinant human neurotrophic factors accelerate colonic transit and relieve constipation in humans. Gastroenterology 119, 41–50 10889153 10.1053/gast.2000.8553

[55] Varendi K., Mätlik K., and Andressoo J. O. (2015) From microRNA target validation to therapy: lessons learned from studies on BDNF. Cell. Mol. Life Sci. 72, 1779–1794 25601223 10.1007/s00018-015-1836-z PMC4412727

[56] Cazorla M., Prémont J., Mann A., Girard N., Kellendonk C., and Rognan D. (2011) Identification of a low-molecular weight TrkB antagonist with anxiolytic and antidepressant activity in mice. J. Clin. Invest. 121, 1846–1857 21505263 10.1172/JCI43992 PMC3083767

[57] Visanji N. P., Brooks P. L., Hazrati L. N., and Lang A. E. (2013) The prion hypothesis in Parkinson's disease: Braak to the future. Acta Neuropathol. Commun. 1, 2 24252164 10.1186/2051-5960-1-2 PMC3776210

[58] Lema Tomé C. M., Tyson T., Rey N. L., Grathwohl S., Britschgi M., and Brundin P. (2013) Inflammation and α-synuclein's prion-like behavior in Parkinson's disease: is there a link? Mol. Neurobiol. 47, 561–574 22544647 10.1007/s12035-012-8267-8 PMC3589652

[59] Chauhan A., and Jeans A. F. (2015) Is Parkinson's disease truly a prion-like disorder? An appraisal of current evidence. Neurol Res. Int. 2015, 345285 25653875 10.1155/2015/345285 PMC4310229

[60] Beach T. G., Adler C. H., Dugger B. N., Serrano G., Hidalgo J., Henry-Watson J., Shill H. A., Sue L. I., Sabbagh M. N., Akiyama H., and Arizona Parkinson's Disease Consortium (2013) Submandibular gland biopsy for the diagnosis of Parkinson disease. J. Neuropathol. Exp. Neurol. 72, 130–136 23334596 10.1097/NEN.0b013e3182805c72 PMC3571631

[61] Schapira A. H., and Olanow C. W. (2003) Rationale for the use of dopamine agonists as neuroprotective agents in Parkinson's disease. Ann. Neurol. 53, S149–S157; discussion S157–S149 12666106 10.1002/ana.10514

[62] Hyman C., Hofer M., Barde Y. A., Juhasz M., Yancopoulos G. D., Squinto S. P., and Lindsay R. M. (1991) BDNF is a neurotrophic factor for dopaminergic neurons of the substantia nigra. Nature 350, 230–232 2005978 10.1038/350230a0

[63] Mogi M., Togari A., Kondo T., Mizuno Y., Komure O., Kuno S., Ichinose H., and Nagatsu T. (1999) Brain-derived growth factor and nerve growth factor concentrations are decreased in the substantia nigra in Parkinson's disease. Neurosci. Lett. 270, 45–48 10454142 10.1016/s0304-3940(99)00463-2

[64] Howells D. W., Porritt M. J., Wong J. Y., Batchelor P. E., Kalnins R., Hughes A. J., and Donnan G. A. (2000) Reduced BDNF mRNA expression in the Parkinson's disease substantia nigra. Exp. Neurol. 166, 127–135 11031089 10.1006/exnr.2000.7483

[65] Höglinger G. U., Widmer H. R., Spenger C., Meyer M., Seiler R. W., Oertel W. H., and Sautter J. (2001) Influence of time in culture and BDNF pretreatment on survival and function of grafted embryonic rat ventral mesencephalon in the 6-OHDA rat model of Parkinson's disease. Exp. Neurol. 167, 148–157 11161602 10.1006/exnr.2000.7546

[66] Sun M., Kong L., Wang X., Lu X. G., Gao Q., and Geller A. I. (2005) Comparison of the capability of GDNF, BDNF, or both, to protect nigrostriatal neurons in a rat model of Parkinson's disease. Brain Res. 1052, 119–129 16018990 10.1016/j.brainres.2005.05.072 PMC2581863

[67] Weinreb O., Amit T., Bar-Am O., and Youdim M. B. (2007) Induction of neurotrophic factors GDNF and BDNF associated with the mechanism of neurorescue action of rasagiline and ladostigil: new insights and implications for therapy. Ann. N.Y. Acad. Sci. 1122, 155–168 18077571 10.1196/annals.1403.011

[68] Karamohamed S., Latourelle J. C., Racette B. A., Perlmutter J. S., Wooten G. F., Lew M., Klein C., Shill H., Golbe L. I., Mark M. H., Guttman M., Nicholson G., Wilk J. B., Saint-Hilaire M., DeStefano A. L., Prakash R., et al (2005) BDNF genetic variants are associated with onset age of familial Parkinson disease: GenePD Study. Neurology 65, 1823–1825 16344533 10.1212/01.wnl.0000187075.81589.fd

[69] Nagahara A. H., and Tuszynski M. H. (2011) Potential therapeutic uses of BDNF in neurological and psychiatric disorders. Nat. Rev. Drug Discov. 10, 209–219 21358740 10.1038/nrd3366

[70] Chun H. S., Son J. J., and Son J. H. (2000) Identification of potential compounds promoting BDNF production in nigral dopaminergic neurons: clinical implication in Parkinson's disease. Neuroreport 11, 511–514 10718305 10.1097/00001756-200002280-00017

[71] Yang J., Siao C. J., Nagappan G., Marinic T., Jing D., McGrath K., Chen Z. Y., Mark W., Tessarollo L., Lee F. S., Lu B., and Hempstead B. L. (2009) Neuronal release of proBDNF. Nat. Neurosci. 12, 113–115 19136973 10.1038/nn.2244 PMC2737352

[72] Fredriksson A., Stigsdotter I. M., Hurtig A., Ewalds-Kvist B., and Archer T. (2011) Running wheel activity restores MPTP-induced functional deficits. J. Neural Transm. 118, 407–420 20852902 10.1007/s00702-010-0474-8

[73] Manning-Bog A. B., McCormack A. L., Li J., Uversky V. N., Fink A. L., and Di Monte D. A. (2002) The herbicide paraquat causes up-regulation and aggregation of α-synuclein in mice: paraquat and α-synuclein. J. Biol. Chem. 277, 1641–1644 11707429 10.1074/jbc.C100560200

[74] Vila M., Vukosavic S., Jackson-Lewis V., Neystat M., Jakowec M., and Przedborski S. (2000) α-Synuclein up-regulation in substantia nigra dopaminergic neurons following administration of the parkinsonian toxin MPTP. J. Neurochem. 74, 721–729 10646524 10.1046/j.1471-4159.2000.740721.x

[75] Junn E., and Mouradian M. M. (2012) MicroRNAs in neurodegenerative diseases and their therapeutic potential. Pharmacol. Ther. 133, 142–150 22008259 10.1016/j.pharmthera.2011.10.002 PMC3268953

[76] Nakamura K., Mori F., Kon T., Tanji K., Miki Y., Tomiyama M., Kurotaki H., Toyoshima Y., Kakita A., Takahashi H., Yamada M., and Wakabayashi K. (2015) Filamentous aggregations of phosphorylated α-synuclein in Schwann cells (Schwann cell cytoplasmic inclusions) in multiple system atrophy. Acta Neuropathol. Commun. 3, 29 25990096 10.1186/s40478-015-0208-0 PMC4438578

[77] Krismer F., Jellinger K. A., Scholz S. W., Seppi K., Stefanova N., Antonini A., Poewe W., and Wenning G. K. (2014) Multiple system atrophy as emerging template for accelerated drug discovery in α-synucleinopathies. Parkinsonism Relat. Disord. 20, 793–799 24894118 10.1016/j.parkreldis.2014.05.005 PMC4141743

[78] Zhang L. (2011) Voluntary oral administration of drugs in mice. Nature Protocol Exchange 10.1038/protex.2011.236

[79] Taylor T. N., Caudle W. M., Shepherd K. R., Noorian A., Jackson C. R., Iuvone P. M., Weinshenker D., Greene J. G., and Miller G. W. (2009) Nonmotor symptoms of Parkinson's disease revealed in an animal model with reduced monoamine storage capacity. J. Neurosci. 29, 8103–8113 19553450 10.1523/JNEUROSCI.1495-09.2009 PMC2813143

[80] Kuo Y. M., Li Z., Jiao Y., Gaborit N., Pani A. K., Orrison B. M., Bruneau B. G., Giasson B. I., Smeyne R. J., Gershon M. D., and Nussbaum R. L. (2010) Extensive enteric nervous system abnormalities in mice transgenic for artificial chromosomes containing Parkinson disease-associated α-synuclein gene mutations precede central nervous system changes. Hum. Mol. Genet. 19, 1633–1650 20106867 10.1093/hmg/ddq038 PMC2850613

[81] Li L., Rutlin M., Abraira V. E., Cassidy C., Kus L., Gong S., Jankowski M. P., Luo W., Heintz N., Koerber H. R., Woodbury C. J., and Ginty D. D. (2011) The functional organization of cutaneous low-threshold mechanosensory neurons. Cell 147, 1615–1627 22196735 10.1016/j.cell.2011.11.027 PMC3262167

[82] Waxman E. A., and Giasson B. I. (2008) Specificity and regulation of casein kinase-mediated phosphorylation of α-synuclein. J. Neuropathol. Exp. Neurol. 67, 402–416 18451726 10.1097/NEN.0b013e3186fc995 PMC2930078

[83] Schneider C. A., Rasband W. S., and Eliceiri K. W. (2012) NIH Image to ImageJ: 25 years of image analysis. Nat. Methods 9, 671–675 22930834 10.1038/nmeth.2089 PMC5554542

[84] Lou H., Montoya S. E., Alerte T. N., Wang J., Wu J., Peng X., Hong C. S., Friedrich E. E., Mader S. A., Pedersen C. J., Marcus B. S., McCormack A. L., Di Monte D. A., Daubner S. C., and Perez R. G. (2010) Serine 129 phosphorylation reduces the ability of α-synuclein to regulate tyrosine hydroxylase and protein phosphatase 2A *in vitro* and *in vivo*. J. Biol. Chem. 285, 17648–17661 20356833 10.1074/jbc.M110.100867 PMC2878529

[85] Aranda P. S., LaJoie D. M., and Jorcyk C. L. (2012) Bleach gel: a simple agarose gel for analyzing RNA quality. Electrophoresis 33, 366–369 22222980 10.1002/elps.201100335 PMC3699176

[86] Tehranian R., Montoya S. E., Van Laar A. D., Hastings T. G., and Perez R. G. (2006) α-Synuclein inhibits aromatic amino acid decarboxylase activity in dopaminergic cells. J. Neurochem. 99, 1188–1196 16981894 10.1111/j.1471-4159.2006.04146.x

[87] Pfaffl M. W., Horgan G. W., and Dempfle L. (2002) Relative expression software tool (REST) for group-wise comparison and statistical analysis of relative expression results in real-time PCR. Nucleic Acids Res. 30, e36 11972351 10.1093/nar/30.9.e36 PMC113859

